# Correlation between serum α-Klotho levels and different stages of periodontitis

**DOI:** 10.1186/s12903-023-03099-4

**Published:** 2023-06-08

**Authors:** Can Ni, Dongyu Bao, Fuhua Yan, Bin Chen

**Affiliations:** 1 Department of Periodontology, Nangjing Stomatological Hospital, Affiliated Hospital of Medical School, Nanjing University, Nanjing, 210008 China; 2 Department of Stomatology, Drum Tower Hospital, The Affiliated Hospital of Nanjing University Medical School, Nanjing, 210008 China

**Keywords:** α-Klotho, Stages of periodontitis, Correlation, National Health and Nutrition Examination Survey (NHANES), Cross-sectional study

## Abstract

**Background:**

Periodontitis is an inflammatory disease characterized by inflammation and progressive destruction of periodontal tissues including alveolar bone. α-klotho protein is a multifunctional protein related to age-related diseases, inflammatory diseases, and bone metabolism-related diseases. However, large-sample epidemiological research evidence on the correlation between α-Klotho and the aggravation of periodontitis stages is still lacking.

**Methods:**

Cross-sectional study data of participants aged between 40 and 79 years in the National Health and Nutrition Examination Survey 2013‒2014 were selected and analyzed. The stages of periodontitis of the participants were determined according to the 2018 World Workshop Classification of Periodontal and Peri-implant Diseases. The serum α-Klotho levels in people with periodontitis in different stages were evaluated. Then the correlation between serum α-Klotho levels and different stages of periodontitis was analyzed by multiple linear regression (stepwise regression method).

**Results:**

A total of 2378 participants were included in the study. The serum α-Klotho levels in people with stage I/II, III and IV periodontitis were 896.16 ± 304.84, 871.08 ± 266.42 and 840.52 ± 286.24 pg/mL, respectively. The levels of α-Klotho in people with stage IV periodontitis were significantly lower than those in people with stage I/II and III periodontitis. Linear regression analysis results showed that compared to stage I/II periodontitis, serum α-Klotho levels were significantly negatively correlated with stage III (B ± SE = -37.28 ± 16.00, *95% CI*: -68.66 ~ -25.91, *P* = 0.020) and stage IV (B ± SE = -69.37 ± 16.11, *95% CI*: -100.97 ~ -37.77, *P* < 0.001) periodontitis.

**Conclusion:**

The serum α-Klotho levels were negatively correlated with the severity of periodontitis. With the aggravation of periodontitis stages, the serum α-Klotho levels gradually decreased.

## Background

Periodontitis is a kind of chronic inflammatory disease caused by plaque microorganisms. It is mainly characterized by inflammation and progressive destruction of periodontal tissues including periodontal ligament, cementum and alveolar bone. Periodontitis is not only one of the main causes of tooth loss in adults, but also closely related to general health. Many studies have shown that periodontitis is an important risk factor for diabetes and cardiovascular diseases and is closely related to Alzheimer's disease, chronic kidney disease, respiratory diseases, cancer, metabolic syndrome, osteoporosis, arthritis and so on [1, 2].

The results of epidemiological surveys show that periodontitis is the sixth most common disease in humans. In China, more than 60% of adults suffer from periodontitis in varying degrees, while in the United States, the prevalence of periodontitis is more than 40% [3, 4]. The occurrence and development of periodontitis result from the interaction between bacterial biofilms and host immune response. Oxidative stress, systemic infection or inflammation, abnormal tissue metabolism, cell aging and cell apoptosis can all promote the progression of periodontitis by affecting host immunity or periodontal flora [5, 6]. At the same time, periodontitis can also affect systemic inflammation and metabolism through the blood pathway (bacteria and inflammatory mediators spread to the whole body by entering the blood circulation) [7], intestinal flora pathway [8] and so on, thus affecting general health. However, the molecular mechanism of the association between periodontitis and systemic diseases still needs further study.α-Klotho protein, a multifunctional protein, was first found as an anti-aging protein [9], and later was found to have important functions in anti-oxidative stress, anti-inflammation, regulation of calcium and phosphorus balance, regulation of tissue metabolism, and anti-apoptosis [10, 11]. α-Klotho is mainly expressed in the kidney, and is also expressed in blood vessels, brain, pancreas, thyroid, and skin tissues [12]. A recent study found that α-Klotho can also be expressed in human alveolar bone [13]. α-Klotho has been found to be associated with a variety of diseases, including age-related diseases, inflammatory diseases, and bone metabolism-related diseases, such as Alzheimer's disease, cardiovascular and cerebrovascular diseases, kidney disease, chronic obstructive pulmonary disease, cancer, diabetes, rheumatoid arthritis and osteoporosis. The progression and poor prognosis of these diseases are related to the decreased expression of α-Klotho, while increasing the expression of α-Klotho can show a certain therapeutic effect [10].

Considering the possible role of α-Klotho along with the pathogenesis and characteristics of periodontitis, it is hypothesized that α-klotho protein may also be correlated with periodontitis. Since few studies on the expression and function of α-Klotho protein in people with periodontitis are reported, the relationship between α-Klotho protein and periodontitis is not quite clear. Still, large-sample epidemiological research evidence on the correlation between α-Klotho levels and the stages of periodontitis is still lacking. Therefore, to explore if the exacerbation of periodontitis is related to lower serum α-klotho levels would be a very interesting issue. Hence, this study analyzed the expression levels of serum α-klotho protein in patients with periodontitis in different stages and investigated their correlation based on the 2018 World Workshop Classification of Periodontal and Peri-implant Diseases and the data of the National Health and Nutrition Examination Survey (NHANES).

## Methods

### Study design and population

The cross-sectional study was based on data from the National Health and Nutrition Examination Survey (NHANES), which was conducted by the Centers for Disease Control and Prevention (CDC) and the National Center for Health Statistics (NCHS) in 2-year cycles and examined the health and nutrition condition of a representative U.S. population using a stratified, multistage probability sampling method. All investigations and study procedures were approved by the NCHS Research Ethics Review Board, and written informed consent was obtained from all participants [14]. The original data and more details are publicly available on the NHANES website (https://www.cdc.gov/nchs/nhanes/index.htm).

**Fig. 1:**
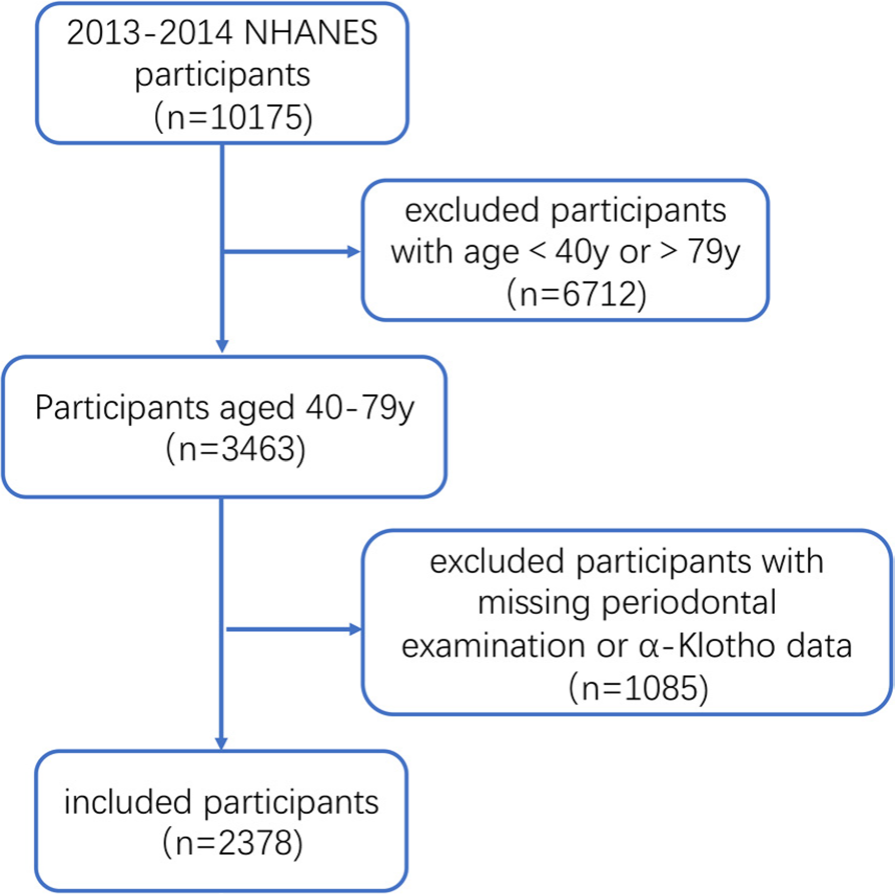
Screening process of study participants

In this study, the 2013–2014 NHANES data were extracted for integration and analysis. Participants aged < 40 or > 79 years and those with missing periodontitis examination data or α-Klotho data were excluded. Figure 1 shows the screening process of the study population.

### Periodontal examination and classification of periodontitis

Full-mouth periodontal examination of study subjects was conducted by trained and licensed dentists. Gingival recession, probing depth (PD) and clinical attachment loss (CAL) at six sites (mesial-buccal, central-buccal, distal-buccal, mesial-lingual, central-lingual, and distal-lingual) in each tooth were recorded, and the number of missing teeth was recorded through dental examination.

Periodontitis classification was determined according to the 2018 World Workshop Classification of Periodontal and Peri-implant Diseases [15]. The periodontitis stage of the participants was evaluated based on PD, CAL, and the number of missing teeth. Periodontitis was diagnosed when CAL ≥ 1 mm in two or more non-adjacent teeth, or ≥ 2 teeth with buccal or lingual CAL ≥ 3 mm and concurrent PD ≥ 3 mm were found. Periodontitis stage: the CAL of the heaviest site was selected first. A CAL of 1–2 mm was defined as stage I, a CAL of 3–4 mm was defined as stage II, and a CAL ≥ 5 mm was defined as stage III. Second, the number of missing teeth was considered (in this study, as the loss of teeth due to caries and periodontitis was difficult to differentiate in a cross-sectional survey, so the missing teeth were simply divided into missing due to dental diseases and missing due to other reasons, and the number of missing teeth due to dental diseases was included in the classification of periodontitis). Stages I and II had no missing teeth, stage III had ≤ 4 missing teeth, and stage IV had ≥ 5 missing teeth. Finally, the stage was adjusted according to the complexity of periodontitis. If the maximum PD in stage II was ≥ 6 mm, it was redefined as stage III, and if the number of remaining teeth in stage III was less than 20 or less than 10 pairs of occlusal teeth, it was redefined as stage IV [4]. Considering that only 81 participants (3.41% of the total participants) were diagnosed with stage I periodontitis, we combined stage I and stage II periodontitis into one category for analysis. Participants were divided into 3 groups according to stages of periodontitis: stages I/II, III, and IV.

### Concentration of Serum α-Klotho

In mobile examination centers (MECs), blood samples of participants with obtained consent were collected in the morning after fasting for at least 9 h [16]. The samples were packaged on dry ice and then stored at -80 °C for further analysis. Serum α-Klotho analysis was conducted by the Northwest Lipid Metabolism and Diabetes Research Laboratory in the Division of Metabolism, Endocrinology, and Nutrition, University of Washington. The concentration of α-Klotho in serum was determined by an ELISA kit (IBL, Japan). All samples were tested in duplicate, and the average value of two tests was used as the final test result.

### Covariate assessment

Some covariates possibly related to periodontitis or α-Klotho were included into analysis [17, 18]. Information on age, sex, race (Mexican American, other Hispanic, non-Hispanic black, non-Hispanic white, other), smoking status (at least 100 cigarettes in life), and alcohol consumption (at least 12 alcohol drinks per year) were collected through household interviews and questionnaires. Body mass index (BMI), blood pressure, total cholesterol (TC), triglyceride (TG), low density lipoprotein (LDL), glycosylated hemoglobin (HbA1c), oral glucose tolerance test (OGTT) 2 h blood glucose, 25-hydroxyvitamin D, white blood cell count and hemoglobin were measured. Systematic disease history including hypertension, diabetes, hyperlipidemia, cardiovascular disease, thyroid disease, osteoporosis, arthritis and cancer, was also recorded. Hypertension was diagnosed by mean systolic blood pressure above 140 mmHg and/or diastolic blood pressure above 90 mmHg, or by self-reported hypertension or use of antihypertensive medication. Diabetes was defined as an OGTT2h blood glucose level higher than 11.1 mmol/L and/or a glycated hemoglobin level higher than 6.5%, self-reported diabetes or current use of antidiabetic drugs. Hyperlipidemia was diagnosed when a total cholesterol level higher than 6.22 mmol/L, a triglyceride level higher than 2.26 mmol/L, and/or a low-density lipoprotein level higher than 4.14 mmol/L, self-reported hyperlipidemia or current use of lipid-lowering drugs. Cardiovascular disease was diagnosed based on a self-reported history of congestive heart failure, coronary heart disease, angina, or heart attack. Thyroid disease, osteoporosis, arthritis, and cancer were diagnosed based on patient feedback on relevant medical history.

### Statistical analysis

GraphPad Prism 9 and SPSS 26.0 software were used for statistical analysis. The differences in characteristics of the study population stratified by periodontal status were compared by one-way ANOVA for continuous variables (recorded as X̅ ± SD) and the chi-square test for categorical variables (recorded as N (%)). Serum α-Klotho levels in patients with periodontitis in different stages were analyzed by one-way ANOVA followed by the Holm-Sidak multiple comparison test. To exclude the interference of possible confounding covariates, the relationship between α-Klotho and periodontitis stage was analyzed by multiple linear regression (stepwise regression) and adjusted for age, gender, race, BMI, smoking, drinking, vitamin D, hemoglobin, white blood cell count, hypertension, diabetes, hyperlipidemia, cardiovascular disease, thyroid disease, osteoporosis, arthritis and cancer. The α-Klotho level was set as the dependent variable, and periodontitis stages and other variables were set as independent variables. Through the stepwise regression method, the nonsignificant independent variables were eliminated and only the independent variables with significant effects were included in the equation, resulting in a locally optimal regression equation. The selected significant independent variables are presented as the unstandardized coefficient (B), standard error (SE), 95% corresponding confidence intervals (*95% CI*) and *P* values. *P* < 0.05 was considered statistically significant.

## Results

### General characteristics

Among total 10,175 participants, 2378 participants aged 40–79 years with complete periodontal examination and serum α-Klotho data were included (Fig. 1). The characteristics of the included participants are shown in Table 1. The overall average age was 56.95 ± 10.74 years, including 47.65% in males and 52.35% in females. According to the 2018 World Workshop Classification of Periodontal and Peri-implant Diseases, 536 participants were diagnosed with stage I/II periodontitis, while 861 and 981 participants were with stage III and IV periodontitis, respectively. There were significant differences in age, gender, race, smoking and alcohol consumption status among different stages of periodontitis. Compared with patients with stage I/II periodontitis, patients with stage III and IV periodontitis had higher blood pressure, total cholesterol, low-density lipoprotein, glycosylated hemoglobin, OGTT 2 h blood glucose, and white blood cell count, lower 25-hydroxyvitamin D levels, and higher proportions of hypertension, diabetes, hyperlipidemia, cardiovascular disease, osteoporosis, and arthritis.

**Table 1:** Characteristics of the study population overall and stratified by periodontal status

	**Stage I/II**	**Stage III**	**Stage IV**	**Total**	***P***
**Number**	536	861	981	2378	
**Age**	51.04 ± 9.08	55.32 ± 10.20	61.62 ± 10.01	56.95 ± 10.74	< 0.001
**Gender**					0.017
Male	227 (42,35)	430 (49.94)	476 (48.52)	1133 (47.65)	
Female	309 (57.65)	431 (50.06))	505 (51.48)	1245 (52.35)	
**Race**					< 0.001
Mexican American	55 (10.26)	166 (19.28)	120 (12.23)	341 (14.34)	
Other Hispanic	30 (5.6)	87 (10.10)	110 (11.21)	227 (9.55)	
Non-Hispanic black	307 (57.28)	354 (41.11)	383 (39.04)	1044 (43.90)	
Non-Hispanic white	49 (9.14)	134 (15.56)	257 (26.20)	440 (18.50)	
Other	95 (17.72)	120 (13.94)	111 (11.31)	326 (13.71)	
**Number of lost teeth**	0.52 ± 1.27	2.21 ± 1.44	15.13 ± 8.53	7.16 ± 8.73	< 0.001
**BMI (kg/m**^**2**^**)**	29.00 ± 6.66	29.64 ± 6.61	29.77 ± 6.95	29.55 ± 6.76	0.096
**Systolic blood pressure (mmHg)**	119.68 ± 15.29	125.47 ± 16.42	130.17 ± 19.68	126.07 ± 18.03	< 0.001
**Diastolic blood pressure (mmHg)**	71.85 ± 10.22	72.51 ± 10.85	70.51 ± 12.40	71.55 ± 11.40	0.001
**TC (mmol/L)**	5.15 ± 1.03	5.12 ± 1.23	4.94 ± 1.06	5.05 ± 1.12	< 0.001
**TG (mmol/L)**	1.36 ± 1.19	1.52 ± 2.50	1.41 ± 0.98	1.44 ± 1.70	0.417
**LDL (mmol/L)**	3.08 ± 0.93	3.16 ± 0.85	2.92 ± 0.92	3.01 ± 0.90	0.025
**HbA1c (%)**	5.64 ± 0.91	5.93 ± 1.15	6.14 ± 1.34	5.95 ± 1.20	< 0.001
**OGTT 2 h blood glucose (mmol/L)**	6.72 ± 2.70	6.73 ± 2.44	7.33 ± 3.58	6.95 ± 2.99	0.017
**25-hydroxyvitamin D (nmol/L)**	76.12 ± 30.06	68.19 ± 27.95	67.06 ± 30.06	69.51 ± 29.52	< 0.001
White blood cell (*10^**9**^**/L)**	6.88 ± 2.07	7.19 ± 2.13	7.32 ± 2.19	7.17 ± 2.15	< 0.001
**Hemoglobin (g/dL)**	14.02 ± 1.38	14.09 ± 1.46	13.79 ± 1.53	13.95 ± 1.48	< 0.001
**Alcohol consumption (≥ 12drinks per year)**	397(78.46)	611(74.24)	631(67.63)	1639(72.46)	< 0.001
**Smoking (≥ 100 cigarettes in life)**	162(30.22)	344(39.95)	592(60.41)	1098(46.19)	< 0.001
**Hypertension**	204(38.06)	411(47.74)	628(64.02)	1243(52.27)	< 0.001
**Diabetes**	67(12.50)	181(21.02)	293(29.87)	541(22.75)	< 0.001
**Hyperlipidemia**	286(53.36)	478(55.52)	596(60.75)	1360(57.19)	0.010
**Cardiovascular disease**	17(3.17)	48(5.59)	131(13.42)	196(8.27)	< 0.001
**Osteoporosis**	21(3.92)	58(6.77)	90(9.19)	169(7.12)	< 0.001
**Arthritis**	136(25.37)	252(29.30)	425(43.46)	813(34.25)	< 0.001
**Cancer**	56(10.45)	74(8.59)	131(13.35)	261(10.98)	0.005
**Thyroid disease**	68(12.69)	100(11.66)	126(12.87)	294(12.39)	0.712
**α-Klotho (pg/mL)**	896.16 ± 304.84	871.08 ± 266.42	840.52 ± 286.24	864.13 ± 284.35	< 0.001

Continuous variables are shown as X̅ ± SD, categorical variables are shown as N(%); The Absent values includes: Alcohol consumption (*n* = 116, 4.88%), Smoking (*n* = 1, < 0.01%), Cardiovascular disease (*n* = 7, < 0.01%), Osteoporosis (*n* = 4, < 0.01%), Arthritis (*n* = 4, < 0.01%), Thyroid disease (*n* = 5, < 0.01%)

### Serum α-Klotho levels in patients with periodontitis in different stages

**Fig. 2:**
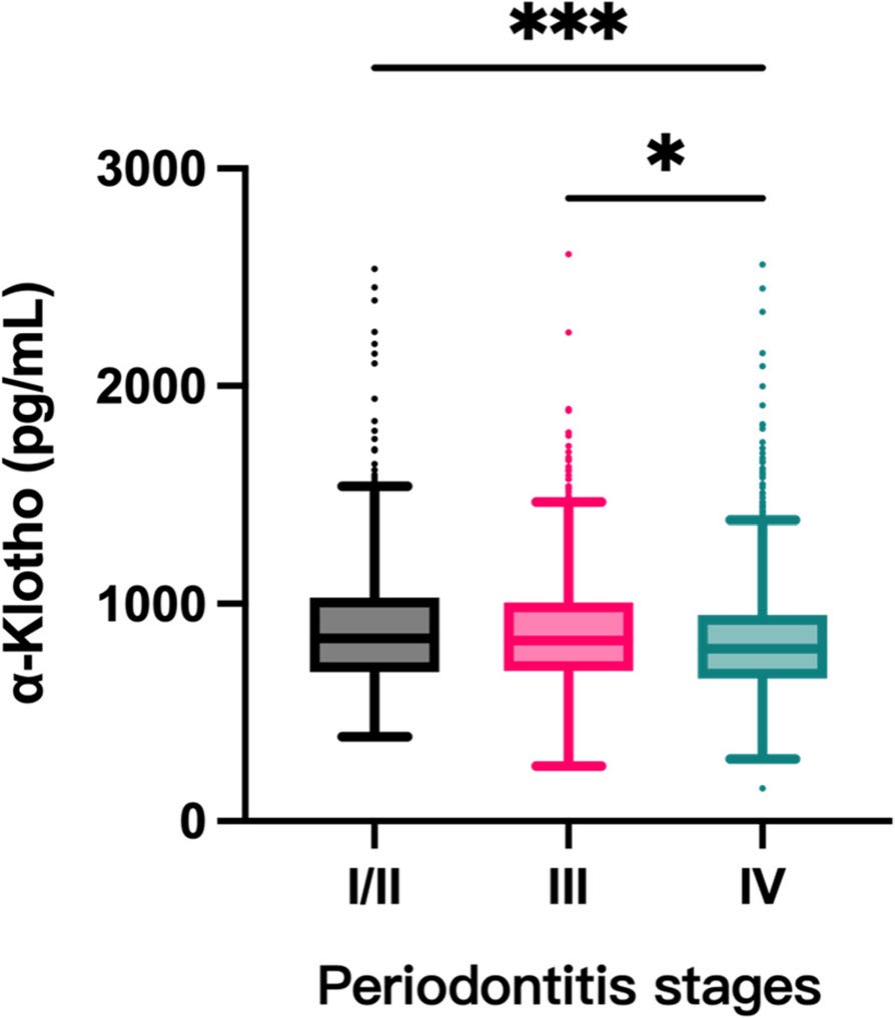
Serum α-Klotho levels in patients with periodontitis in different stages. (*, *P* < 0.05; ***, *P* < 0.001)

There was a significant difference in serum α-Klotho protein levels among patients with stage I/II, stage III, and stage IV periodontitis (896.16 ± 304.84, 871.08 ± 266.42, and 840.52 ± 286.24, respectively) (Table 1). The levels of α-Klotho in the stage IV periodontitis group were significantly lower than those in stage III periodontitis (*P* = 0.04) and stage I/II periodontitis groups (*P* < 0.001) (Fig. 2).

### Correlation between serum α-Klotho levels and different stages of periodontitis

Multiple linear regression (stepwise regression) analysis showed that compared with stage I/II periodontitis, serum α-Klotho levels were significantly negatively associated with stage III periodontitis (B ± SE = -37.28 ± 16.00, *95% CI*: -68.66 to -25.91, *P* = 0.020) and stage IV periodontitis (B ± SE = -69.37 ± 16.11, *95% CI*: -100.97 to -37.77, *P* < 0.001) (Table 2). In this multivariate model, except for periodontitis, other covariates including hyperlipidemia, cardiovascular disease, diabetes, alcohol consumption and white blood cell count were also associated with serum α-Klotho levels.

**Table 2:** Correlation between serum α-Klotho levels and different stages of periodontitis as well as other covariates

Covariates	B	SE	*95%CI*			*P*
			Upper		Lower	
**Periodontitis stages**						
Stage I/II	0(Reference)	0(Reference)	0(Reference)	0(Reference)		0(Reference)
Stage III	-37.28	16.00	-68.66	-5.906		0.020
Stage IV	-69.37	16.11	-100.97	-37.77		< 0.001
**Diabetes**	33.98	14.98	4.62	63.35		0.023
**Hyperlipidemia**	-45.93	12.35	-70.15	-21.72		< 0.001
**Cardiovascular disease**	-46.10	22.32	-89.87	-2.34		0.039
**Alcohol consumption**	-46.65	13.59	-73.29	-20.00		< 0.001
**White blood cell**	-8.60	2.83	-14.15	-3.06		0.002

Dependent covariate: serum α-Klotho levels; Independent covariates: periodontitis stages, age, gender, race, BMI, smoking, drinking, vitamin D, hemoglobin, white blood cell, hypertension, diabetes, hyperlipidemia, cardiovascular disease, thyroid disease, osteoporosis, arthritis, cancer, among which only significant independent variables are shown in the table

B: unstandardized coefficient; SE: Standard error; *95% CI*: 95% corresponding confidence intervals

## Discussion

The severity of periodontitis is closely related to general health. This study found that compared with stage I/II periodontitis patients, stage III and IV periodontitis patients had higher blood pressure, blood lipids, blood glucose and white blood cell count but lower 25-hydroxyvitamin D levels, suggesting an increased risk of hypertension, hyperlipidemia, diabetes, osteoporosis, arthritis, and other diseases. This is also consistent with the increased prevalence of these diseases in the study. Thus, periodontitis may be a risk factor for the above systemic diseases.

The results of this study also showed that serum α-Klotho levels were negatively correlated with the severity of periodontitis, indicating that decreased serum α-Klotho levels may increase the risk of periodontal tissue destruction and aggravate the stage of periodontitis. Some previous studies also support this speculation. A small sample clinical observational study showed that the expression of α-Klotho in gingival crevicular fluid and gingival tissue of periodontitis patients was significantly lower than that of periodontal healthy subjects [19]. Another study showed that serum α-Klotho levels in patients with moderate to severe periodontitis were lower than those in healthy or mild-periodontitis populations [20]. Preclinical intervention studies have shown that α-Klotho gene mutation or gene knockout mice have abnormal periodontal tissue morphological changes, including narrow periodontal space, disordered fiber arrangement and amorphous structure in the periodontal ligament [21]. Specific knockout of the Klotho gene in Osx + mesenchymal progenitor cells can cause abnormal alveolar bone remodeling, aggravate periapical lesions and inhibit alveolar bone repair [13].

Although the mechanisms underlying the association between α-Klotho expression and periodontitis remain unclear, it may be achieved through the following mechanisms:

1) Regulation of calcium-phosphorus balance and vitamin D homeostasis. Α-Klotho is a type-I single-chain transmembrane protein that can act as a coreceptor of fibroblast growth factor 23 (FGF23) and promote the binding of FGF23 with its receptor [22]. FGF23, which is produced by osteocytes in response to phosphate, can increase urinary phosphate excretion after bonding with α-Klotho, lower serum active vitamin D levels by inhibiting the synthesis of active vitamin D and suppress the secretion of parathyroid hormone [17]. Thus, the FGF23-α-Klotho axis plays a vital role in regulating and maintaining calcium-phosphorus balance as well as vitamin D homeostasis. Recent studies have found that α-Klotho can also be expressed in bone tissues, including alveolar bone, suggesting that it may participate in the regulation of local bone metabolism [13]. The reduction in the expression of α-Klotho may induce resistance to the FGF23 response in kidneys, disrupt phosphate excretion and result in an increase in phosphate level, subsequently elevating FGF23 production. The upregulation of FGF23 levels can suppress the active vitamin D levels, leading to increased parathyroid hormone secretion and further enhanced FGF23 levels as well as further depressed α-Klotho levels. This feedback loop ultimately accelerates the calcium-phosphorus imbalance and disease formation [17]. In this study, the 25-hydroxyvitamin D levels were lower in periodontitis at more severe stages, which was consistent with the decreased α-Klotho levels, indirectly suggesting that lower α-Klotho levels may worsen periodontitis by disrupting the calcium-phosphorus balance and vitamin D levels.

2) Regulation of osteogenesis and osteoclast activity. α-Klotho can play a role in promoting bone formation during alveolar bone repair and inhibit osteoclast activity by regulating the TNF signaling pathway and RANKL expression [13]. Another study also confirmed that soluble α-Klotho could promote osteogenic differentiation of osteoblasts by regulating early growth response protein 1 (EGR-1) [23]. However, some other studies reported the opposite effects of α-Klotho on osteogenesis. Zhang et al. found that Klotho protein could inhibit the osteogenic differentiation of humam bone marrow stem cells (hBMSCs) through the FGFR1/ERK signaling pathway [24]. Zhu et al. demonstrated that α-Klotho released from HK-2 cells attenuated the osteogenic differentiation of renal interstitial fibroblasts by inactivating the Wnt-β-catenin pathway [25]. Specific deletion of Klotho in osteocytes led to increased bone formation and bone mass, while overexpression of klotho in osteoblasts suppressed osteogenesis and mineralization [26]. Thus, α-Klotho seems to have dual functions in osteogenesis and osteoclast activity. How α-Klotho regulates the bone formation and resorption in periodontitis is still unclear and is worthy of further study.

3) Anti-oxidative stress. Under H2O2-induced oxidative stress conditions, α-Klotho can not only inhibit oxidative stress and apoptosis of periodontal ligament stem cells by regulating uncoupling protein 2 (UCP2) [19], but also protect the cell viability and osteogenic differentiation ability of periodontal ligament stem cells by maintaining mitochondrial function and antioxidant capacity [27]. In monocytes stimulated by lipopolysaccharide (LPS), α-Klotho could reduce the amounts of reactive oxygen species and inhibit cellular oxidation [28]. Erythropoietin receptor (EpoR) may be a downstream effector participating in the antioxidative activity of α-Klotho, as the cytoprotective effect of α-Klotho can be partially abrogated by deleting EPOR [29]. Moreover, the insulin/insulin-like growth factor-1 pathway, PI3K/AKT-mediated phosphorylation of FoxO proteins, the Nrf2 pathway and the NLRP3 inflammasome may also be involved in the antioxidative stress mechanism of α-Klotho [29].

4) Anti-inflammation. α-Klotho has a good anti-inflammatory function, as it was reported that α-Klotho could induce the degradation of Toll-like receptor 4 [30], reduce the levels of inflammatory cytokines and enhance the expression of the anti-inflammatory factor IL-10 in LPS-stimulated monocytes [28]. An in-vivo study showed that α-Klotho could also significantly reduce the levels of inflammatory factors such as IL-6, IL-1β and TNF-α in the serum of mice with unilateral renal ischemia–reperfusion injury [31]. Downregulation of renal Klotho expression can induce the exacerbation of inflammation in the kidneys in diabetic mice [29]. Another study proved that α-Klotho was significantly associated with well-recognized inflammatory biomarkers, such as C-reactive protein and white blood cell count [32], which was in accordance with the finding that the white blood cell count showed a negative association with α-Klotho level in this study. Thus, these studies indicated the anti-inflammatory role of α-Klotho and explained why some inflammatory disorders are usually accompanied by lower α-Klotho levels, such as rheumatoid arthritis, diabetes, and diabetic nephropathy [33], and, in this study, periodontitis.

5) Regulation of autophagy. Autophagy is an important mechanism for the maintenance of tissue stability. Insufficient or excessive autophagy causes cellular homeostasis disorders, leading to the occurrence or development of diseases. α-Klotho can play a dual role in regulating autophagy in different physiological or pathological conditions, adjusting autophagy to a moderate level, thus showing potential preventive and therapeutic effects on diseases [10]. Therefore, the decrease or deficiency of α-Klotho may lead to the accumulation of reactive oxygen species and inflammatory factors in tissues, disorder of cell autophagy, increase in apoptosis and dysfunction of bone metabolism, subsequently aggravating the progression of periodontitis. However, considering that the current research evidence about the potential mechanisms underlying the relationship between α-Klotho protein and periodontitis is mostly indirect, more efforts are still needed to further investigate and prove these mechanisms.

In this study, except for periodontitis, serum α-Klotho levels were also significantly associated with diabetes, hyperlipidemia, and cardiovascular disease, which was consistent with the results of previous clinical studies. Studies have found that serum α-Klotho levels are correlated with both type-I and type-II diabetes [34, 35]. Another clinical study also reported that the level of serum α-Klotho was associated with the prevalence of cardiovascular disease [36]. Low levels of serum α-Klotho increase the risk of cardiovascular disease and are negatively correlated with lipid metabolism indicators such as triglycerides and total cholesterol [37]. Considering that periodontitis is related to these systemic diseases and that both are also associated with serum α-Klotho levels, it can be speculated that α-Klotho may be involved in mediating the interaction between periodontitis and diabetes, cardiovascular diseases and other diseases, which may be a new pathway to link periodontal diseases with systemic systems. However, this conjecture has not been confirmed thus far, and it is worthy of further study.

In conclusion, serum α-Klotho levels are closely related to the severity of periodontitis, and with the progression of periodontitis stages, serum α-Klotho levels gradually decrease. Therefore, serum α-Klotho may be a potential marker for predicting the severity and risk of periodontitis and may also be a potential treatment target for periodontitis. However, this study also has some limitations. First, since the number of lost teeth in this study cannot exclude the number of teeth lost due to caries, there may be some bias in the periodontitis classification. Second, based on a cross-sectional study, this study can only determine the correlation between serum α-Klotho levels and periodontitis stages, the causal relationship between them cannot be determined. In addition, although many confounding factors were included as covariates in this study for analysis, there may still be other potentially relevant confounding factors that were not included. Therefore, the relationship between α-Klotho and periodontitis also needs to be further verified by prospective randomized controlled trials with large samples.

## Conclusion

The results of this study highlighted the association between α-Klotho and periodontitis, and lower serum α-Klotho levels were correlated with more severe stages of periodontitis. This study provides new insight into the pathogenetic mechanism of periodontitis and suggests the possible application of α-Klotho in risk prediction and therapy of periodontitis.

## Data Availability

The original data are available on the website of NHANES (https://www.cdc.gov/nchs/nhanes/index.htm) and the processed data in this study are available from the corresponding author on reasonable request.
